# Identification of interventions to improve patient experienced quality of care in transitions between healthcare settings: a scoping review

**DOI:** 10.1186/s12913-024-11609-5

**Published:** 2024-09-30

**Authors:** Natasia Hindsbak, Lars Morsø, Dorte Hvidtjørn, Sisse Walløe

**Affiliations:** 1 Odense University Hospital, Odense, Denmark; 2 University of Southern Denmark, Odense, Denmark; 3 Århus University Hospital, Århus, Denmark; 4 Næstved, Slagelse, and Ringsted Hospitals, Slagelse, Denmark

**Keywords:** Patient experience, Scoping review, Healthcare transitions, Interventions, Care coordinators, Integrated care

## Abstract

**Background:**

Transitions in healthcare settings can be a challenge for patients and they express a need for guidance and support to cope with these transitions. The aim of this scoping review was to investigate if interventions can improve patients’ experiences when transitioning between healthcare settings.

**Methods:**

This review was conducted following the Johanna Briggs Institute’s methods and reported according to the PRISMA-ScR Checklist. Included articles were published and peer-reviewed, and reported qualitative and quantitative findings on patient experiences with interventions when transitioning between healthcare settings. The search was conducted in May 2024 in Medline Ovid, Embase Ovid, and Cinahl.

**Results:**

Twenty-three studies were included. Factors extracted from the studies were: author(s), year of publication, country of origin, study design, theoretical methods, population description, intervention, phenomena of interest(s), and key findings. There has been an increase in published studies on the subject in the last few years, and most of the included studies originated from Western countries. Most studies were quantitative, primarily RCTs, and the theoretical methods were thus mainly statistical analysis. The study populations were found to be heterogeneous. The interventions were categorized: care coordinator, program, integrated care, online communication platform, coaching, discharge care plan, and miscellaneous interventions.

**Conclusions:**

Overall, interventions were found to improve the patient experience. Centralization of healthcare has increased the number of transitions, and patients express that the coordination of healthcare transitions can be improved. This review’s findings should be used alongside other research on interventions’ effect on factors like hospital readmissions and mortality to determine the optimal intervention to implement.

**Supplementary Information:**

The online version contains supplementary material available at 10.1186/s12913-024-11609-5.

## Introduction

Navigating healthcare services and transitions can be a great challenge to patients and their families [1–6]. They experience care pathways as fragmented [7] and unsafe [8], and find it difficult to access relevant care leaving patients with unmet needs [9]. This is widely reflected in quality indicators, where patients’ experiences of navigation and coordination are included domains [10–18]. When asking patients about their experience of transitioning between healthcare settings, they express a great need for guidance and support both from their close relatives and caregivers, as well as from healthcare professionals [19–23]. One of the advantages of well-planned and integrated care is the enablement of good patient experiences of transitions between healthcare settings [24]. Patient experienced quality in healthcare is also an indicator of clinical quality and safety for patients and therefor relevant as primary outcome of interventions [11]. However, little is known about how to facilitate coherent healthcare services that can help improve patients’ experiences in these transitions. Some reviews have studied the effects of various interventions to improve patient experience in healthcare transitions.

In a literature review including 37 studies Yu et al. found that case management models seemed to reduce hospital readmissions and emergency department visits [25]. However, they found that case management interventions did not significantly affect patient satisfaction in the three included studies using validated satisfaction questionnaires [25]. Furthermore, in a systematic review of the effects of discharge interventions, Braet et al. described a reduction in readmissions within three months after discharge as the primary outcome [26]. Besides reducing readmissions, they found increased patient satisfaction as a secondary outcome in the intervention groups in five out of six studies [26]. Likewise, Davis et al. saw an increase in patient satisfaction or experience in six out of seven studies in a review of nurse-led services for patients with chronic disease experiencing transitions in healthcare settings [27]. Their primary outcome was continuity of care conceptualized broadly by combining hospital admissions and readmissions, patient experiences, and improvements in symptoms and lifestyle [27]. The concept of patient experience was unfolded more by Tan et al. in their qualitative systematic review of cancer patients’ experiences with navigation programs. They described three general themes: Emotional empowerment, knowledge empowerment, and bridging the gaps [28]. They concluded that patients’ experiences with facing the challenges of cancer were improved by the support of patient navigators [28]. Contrary to this, Jesus et al. were more hesitating in their conclusions due to low evidence quality in included studies of patients’ experiences of transitional care interventions [29]. For most included studies in the systematic review, patient experience of care was improved, however, all studies had risk of bias [29].

To our knowledge, no previous reviews comprehensively illuminate how interventions to improve transitions in healthcare settings influence adult patients’ experiences of quality as primary outcome and/or phenomena of interest. This leaves us with a knowledge gap when developing an intervention to improve healthcare pathways with transitions between settings when the primary outcome for evaluation is patient experience of quality in care. To be able to plan interventions to improve patient experiences of transitions in healthcare settings, it is necessary to review relevant primary studies. Therefore, the aim of this scoping review was to investigate if interventions can improve patients’ experiences when transitioning between healthcare settings.

## Methods

This scoping review was framed by the Johanna Briggs Institute (JBI) methods for scoping reviews to discover research gaps [30]. The methods are based on Arksey and O’Malley’s original framework from 2005 and the revision by Levac and colleagues in 2010, on which JBI’s newest version from 2020 is based [30]. Furthermore, this scoping review was conducted and reported according to the Preferred Reporting Items for Systematic Reviews and Meta-Analyses extension for Scoping Reviews (PRISMA-ScR) Checklist [31].

The first steps of the review were performed in conjunction with a scoping review to explore the concept of patient experience of healthcare transitions (see protocol at OSF | The Exercise First Research Program). However, the in- and exclusion criteria for study selection were targeted to the aim of the current review (see Table 1). The research question “Can interventions improve patients’ experiences of quality in care when transitioning between healthcare settings?”, definitions, and criteria were predefined using the SPIDER model [32] and can be seen in Table 1. Furthermore, we restricted our search to begin from year 2000 as expert knowledge in our group led us to believe that focus on integrated care emerged around the early 2000s.

**Table 1:** Research question defined by the SPIDER-model

		Concepts	Inclusion criteria	Exclusion criteria
S	Setting	Transitions in healthcare settings	Patient movement between at least 2 healthcare settings (municipality, GP, hospital)	Single settings such as “In the primary care setting, at the hospital etc.”
PI	Phenomenon of Interest	Patient-experienced quality	Patients’ experiences were accounted for or assessed	Only healthcare personnel or relatives’ experiences were accounted for.
D	Design	Qualitative and quantitative clinical research designs.	Studies reporting outcomes of interventions	Purely observational or explorative studies. Feasibility studies, study protocols, reports.
E	Evaluation	PREMs, patient accounts, narratives, attitudes, perspectives, and experiences of quality.	Patient experience as an account of what occurred in the encounter with healthcare provision [12, 29].	Patient satisfaction [30].
R	Research Type	Published, peer-reviewed research reporting original data	Peer-reviewed, published reporting original data.	Conference abstracts and meeting notes. Syntheses and reviews.

### Search and selection of included studies

A full search of literature was performed on 7 December 2021 in the databases Medline Ovid, Embase Ovid, and Cinahl and updated 27 May 2024 (Appendix 1). The search strategy is available in detail at OSF | The Exercise First Research Program along with reflections on the in-/exclusion of studies reporting on patient satisfaction versus patient experience. The two terms are sometimes used interchangeably [15], but we only included studies in which the patient experience was unfolded beyond the narrowest understanding of satisfaction [15]. Furthermore, we only included studies in English and the Scandinavian languages.

We used Covidence to manage the screening process, and the program proved effective in identifying duplicates [33]. In total, seven reviewers participated in the screening process, but all studies were screened by at least two independent reviewers. Early in the screening process, a consensus meeting was held to ensure consistency. In the initial scoping review exploring the concept of patient experience of healthcare transitions, it became clear that the number of included articles would be too great to make a meaningful synthesis. Consequently, it was suggested to make a separate scoping review focusing on the association between interventions and patient-experienced quality of care in transitions in healthcare settings.

### Extracting the evidence

Data were extracted using a template inspired by both Covidence’s standard template for reviews and JBI’s list of relevant topics to include [30, 34]. We worked iteratively with the extraction template throughout the extraction process, to ensure fit with the included articles and our aim. The final extraction template can be seen in Appendix 2. The factors that we ended up using from the extraction were: author(s), year of publication, country of origin, study design, theoretical methods, population description, intervention, phenomena of interest(s), and key findings.

### Data charting

To summarize the identified literature, study characteristics are presented with focus on intervention details and categorized by intervention type, e.g. care coordinator, integrated care, program. Furthermore, the effects of interventions to improve patients’ experiences in healthcare transitions are summarized and described graphically and narratively [30].

## Results

**Fig. 1:**
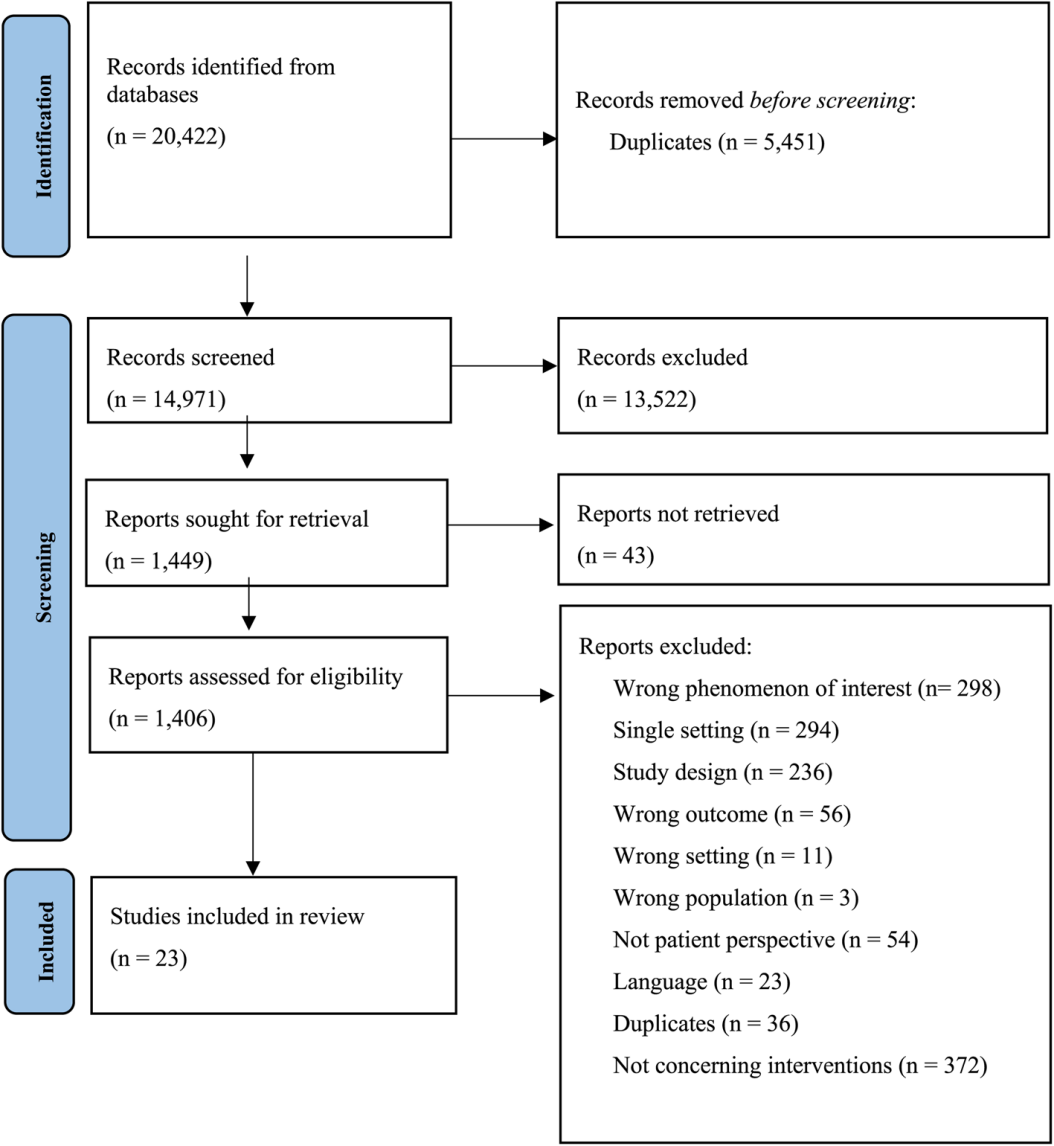
PRISMA Flowchart

The study selection is described in the PRISMA flowchart in Fig. 1. We included 23 studies reporting outcomes of interventions which were relevant to the aim of this review. The 372 reviewed studies which reported on patient-experienced quality in healthcare transitions, but did not concern interventions are described in another scoping review [35].

### Characteristics of the studies

**Fig. 2:**
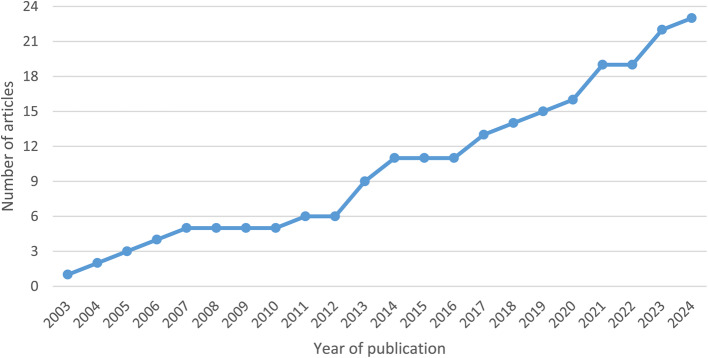
Graph depicting the number of published articles on interventions’ influence on patient experience in transitions between healthcare settings in 2003–2024 cumulated

As seen in Fig. 2 there has been an increase in the number of published studies researching patient experience in transitions in healthcare settings, from less than one per year between 2003 and 2012, to up to three per year between 2013 and 2024. Thirteen studies originate from North America, Denmark, and Sweden. We identified five studies from the US [36–40], three studies from Canada [41–43], three studies from Sweden [44–46], and two studies from Denmark [47, 48]. Only one study from each of the three continents of South America, Asia, and Australia was identified [49–51]. The rest of the identified studies originated from other European countries [52–58]. The characteristics of the studies can be seen in Table 2.

**Table 2:** The characteristics of the included articles on interventions’ impact on patient experience when transitioning between healthcare settings

Year	Authors	Country of origin	Study design	Theoretical approach	Population^b^
2003	Nielsen et al. [47]	Denmark	RCT^a^	Statistical analysis	Cancer-patients
2004	Byng et al. [52]	United Kingdom	RCT	Statistical analysis	Patients with long-term mental illnesses
2005	Preen et al. [51]	Australia	RCT	Statistical analysis	Patients with chronic cardio-respiratory disease
2006	Parry et al. [39]	USA	Qualitative study	Analytical induction, deduction, and negative case comparison	Chronically ill elderly adults
2007	Kautz et al. [37]	USA	Cohort study	Statistical analysis	Patients, who had total knee arthroplasty due to osteoarthritis
2011	Koh et al. [38]	USA	Cohort study	Statistical analysis	Breast-cancer patients
2013	Berglund et al. [44]	Sweden	RCT	Statistical analysis	Elderly people
2013	Boult et al. [36]	USA	RCT	Statistical analysis	Elderly patients
2013	Smidth et al. [48]	Denmark	RCT	Statistical analysis	People living with chronic obstructive pulmonary disease
2014	Schöttle et al. [56]	Germany	Cohort study	Statistical analysis	Patients with schizophrenia and/or bipolar disorder
2014	Wagner et al. [40]	USA	RCT	Statistical analysis	Cancer-patients
2017	Röttger et al. [54]	Germany	Cross-sectional study	Statistical analysis	People with chronic diseases
2017	Scherz et al. [55]	Switzerland	RCT	Statistical analysis	Cancer-patients
2018	Thomson et al. [57]	United Kingdom	Qualitative study	Thematic content analysis	People with depression
2019	Westman et al. [46]	Sweden	Cohort study	Statistical analysis	Cancer-patients
2020	Hu et al. [50]	China	RCT	Statistical analysis	Kidney-transplant recipients
2021	Espinel-Flores et al. [49]	Brazil, Chile, Colombia, Mexico, and Uruguay	Quasi-experimental study	Statistical analysis	People with chronic diseases
2021	Hallgren et al. [45]	Sweden	Qualitative study	Content analysis	People who need emergency care
2021	Jepma et al. [53]	Netherlands	Qualitative study	Thematic content analysis	Fragile, elderly heart-patients
2023	Gavaldà-Espelta et al. [58]	Spain	Quasi-experimental clinical trial	Statistical analysis	People with chronic diseases who are social-dependent
2023	Petrovic et al. [41]	Canada	RCT	Statistical analysis	Cancer-patients
2023	Markle-Reid et al. [43]	Canada	RCT	Statistical analysis	Older adults living with stroke
2024	Chaukos et al. [42]	Canada	Qualitative study	Thematic content analysis	HIV-patients

^a^Randomized controlled trial

^b^Populations are described with the terms and definitions used in the original articles

Most of the included studies used quantitative methods. Eleven were randomized controlled trials (RCT) [36, 40, 41, 43, 44, 47, 48, 50–52, 55] and four were cohort studies [37, 38, 46, 56]. Additionally, three included studies used other quantitative methods, two were quasi-experimental studies [49, 58] and one was a cross-sectional study [54]. Finally, five included studies used qualitative methods [39, 42, 45, 53, 57].

Most of the included studies used statistical analysis [36–38, 40, 41, 43, 44, 46–52, 54–56, 58] reflecting their quantitative methods. The studies using qualitative methods had several different theoretical approaches ranging from use of analytical induction, deduction, and negative case comparison [39] and thematic content analysis [42, 53, 57], to content analysis [45].

The populations of the studies were quite diverse, but all were limited to a single or specific population. Six studies included cancer patients [38, 40, 41, 46, 47, 55], six studies included patients with chronic diseases [39, 48, 49, 51, 54, 58], four studies looked at elderly people [36, 39, 43, 44, 53], and three researched patients with mental illnesses [52, 56, 57]. Furthermore, one study population consisted of HIV-patients [42], another of people who needed emergency care [45], one study researched patients who had a total knee arthroplasty due to osteoarthritis [37], and the last study included kidney transplant recipients [50].

### The interventions and their influence on patient-experience

Of the 23 included studies in this scoping review only three studies did not find a positive effect on the patient experience in transitions across healthcare settings when an intervention was conducted [37, 41, 52]. The remaining 20 articles found a positive effect on patients’ experience. The studies’ interventions can be divided into seven intervention categories: (1) Care coordinator, (2) Program (an intervention with multiple components), (3) Integrated care, (4) Online communication platform, (5) Coaching, (6) Discharge care plan, and (7) Miscellaneous interventions (see Table 3).

**Table 3:** The included studies’ interventions and their effect on patient experience in transitions between healthcare settings

Study	Intervention	Positive effect on patient experience	Key findings related to patient experience
Berglund et al. 2013 [44]	Care coordinator	Yes	The intervention group perceived higher quality in care planning knowledge of whom to contact
Koh et al. 2011 [38]	Care coordinator	Yes	Patients in care coordinator program had high satisfaction with services
Scherz et al. 2017 [55]	Care coordinator	Yes	Case management group increased in score for care provided in accordance with chronic care model
Wagner et al. 2014 [40]	Care coordinator	Yes	Navigator improved patient experience with cancer care and involvement
Westman et al. 2019 [46]	Care coordinator	Yes	Improvement of information, involvement, and care coordination after introduction of navigator
Boult et al. 2013 [36]	Program	Yes	Quality of chronic care was significantly higher with intervention.
Byng et al. 2004 [52]	Program	No	No improvement in satisfaction nor reduction in unmet needs
Hu et al. 2020 [50]	Program	Yes	Improved discharge readiness, transitional care quality and satisfaction with transitional care services in intervention group
Jepma et al. 2021 [53]	Program	Yes	Participants appreciated care continuity and supportive networks
Nielsen et al. 2003 [47]	Program	Yes	Programme bettered cooperation and reduced patients’ feelings of being left in limbo
Röttger et al. 2017 [54]	Program	Yes	Coordination rated better in disease management program
Smidth et al. 2013 [48]	Program	Yes	Significant improvement in chronic care management in intervention group
Thomson et al. 2018 [57]	Program	Yes	Better flexibility and access experienced by patients with collaborative care approach
Markle-Reid et al. 2023 [43]	Program	Yes	Differences favouring intervention group for Person-Centred Coordinated Care Experiences Questionnaire
Hallgren et al. 2021 [45]	Integrated care	Yes	Collaborative healthcare leads to more efficient care
Kautz et al. 2007 [37]	Integrated care	No	No effect of integrated delivery system membership on patient-perceived coordination of care
Schöttle et al. 2014 [56]	Integrated care	Yes	Patients more satisfied with integrated care model than with previous treatment
Chaukos et al. 2024 [42]	Integrated care	Yes	Coordinator (fellow) built trust and effectively implemented care plans supporting patients in engagement in treatment.
Gavaldà-Espelta at al. 2023 [58]	Online communication platform	Yes	Improved treatment adherence and reduced caregiver burden after intervention
Petrovic et al. 2023 [41]	Online communication platform	No	No effect of intervention on continuity of care
Parry et al. 2006 [39]	Coaching	Yes	Patients experienced enhanced self-management and sense of safety and mastery
Preen et al. 2005 [51]	Discharge care plan	Yes	Satisfaction with discharge care planning greater in intervention group
Espinel-Flores et al. 2021 [49]	Miscellaneous interventions	Yes	Improved cross-level continuity of care after implementation of interventions

#### Care coordinator

In five of the included studies, the intervention was a care coordinator [38, 40, 44, 46, 55]. All five studies found a significant improvement in patient experience in patients who were helped by a care coordinator in their transition between healthcare settings, compared to patients without a care coordinator. However, one study found no significant difference in the patient-experienced quality of life [40].

#### Program

In nine of the studies, the interventions were programs [36, 43, 47, 48, 50, 52–54, 57]. The programs were very different in character, but all of them consisted of a variety of interventions that were joined together in a program. As an example, one of the study’s programs consisted of interventions before, during, and after admission for patients who had received a kidney through transplantation. The program consisted of four phases where the patient was consecutively informed about the process ahead and thereby kept prepared for the next step in the process to receive a kidney [50].

In several of the programs, some sort of coordination of patient care by a healthcare professional, such as a nurse, general practitioner (GP), or care coordinator was prevalent. In one of the studies, the program consisted of home-based assessment of patients’ needs and goals, evidence-based care planning, proactive monitoring, care coordination, transitional care, coaching for self-management, caregiver support, and access to community-based services [36].

Seven out of eight of the included studies with a program as intervention found an improvement in patient experience [36, 43, 47, 48, 50, 53, 54, 57]. One study found that the intervention had a different influence on different patient groups, but that the intervention overall gave a better patient experience in transitions between healthcare settings [53]. Only one study with a program as the intervention found no significant difference in patient experience [52].

#### Integrated care

In four studies, the interventions in transitions between healthcare settings were some sort of integrated care [37, 42, 45, 56] defined as a care model where all treatments were gathered under one organization to ensure better communication between different healthcare providers. In one study the intervention consisted of assertive community treatment (ACT) [56], which is a way to organize all treatments under the same organization instead of a care coordinator and is often used in psychiatry [59]. In three of the studies, they found that the intervention improved patient experience [42, 45, 56], while the last study could not find a significant difference between patients who had received the intervention, and patients who had not [37].

#### Online communication platform

In two of the studies a form of online communication platform was used between healthcare providers [41, 58]. In one of the studies the platform was used as a way for the primary care provider to consult with a cancer-specialist [41]. This study could not find any significant effects on the patient experience of continuity of care but did find a significantly lower measure of anxiety in patients from the intervention group compared to those in the control group. In the other study, the platform was used for communication between the healthcare system and the social care system, to improve communication between these two systems [58]. This study found that patients’ quality of life increased significantly over time after the intervention was implemented [58].

#### Coaching

One study looked at coaching as an intervention in transitions between healthcare settings and found a very positive effect on patient experience [39].

#### Discharge care plan

In one study the intervention consisted of a thorough and individualized discharge plan, that was sent from the discharging hospital department to the patients’ GPs [51]. The study found a significant positive effect on patient experience – both in patients’ perceptions of quality of life and patients’ satisfaction with discharge [51].

#### Miscellaneous interventions

The last study included in this scoping review investigated the effect of a range of diverse interventions from five South American countries [49]. In the study, they found that it was possible to do a combined analysis of the effect of the interventions, despite their diversity [49]. They concluded that it was the fact that an intervention was conducted that had positive effects on the patient experience in transitions in healthcare settings [49].

## Discussion

We investigated if interventions can improve patients’ experiences when transitioning between healthcare settings. Overall, we found that interventions can have positive effects on patients’ experiences with transitions between healthcare settings. Improvement interventions seem to help patients navigate between healthcare settings, and patients express better experiences with transitioning between settings when an intervention is conducted. Our findings are supported by results from a meta-qualitative study investigating patient experience with an array of different interventions in transitions in healthcare settings [60]. Here, they found that the interventions gave patients a sense of support and self-empowerment which in turn facilitated patients’ ability to navigate transitions [60]. Likewise, patient navigation for cancer patients improves care satisfaction and reduces time from screening to diagnosis, and hospital readmissions [61].

Healthcare systems have in many developed countries gone through a drastic change during the last 50 years, going from being decentralized back to being centralized [62, 63]. This has both changed the organization of the healthcare system fiscally and administratively [63] as well as centralized the care into fewer, bigger, and more specialized healthcare settings [64]. Centralization and specialization of healthcare have entailed an increased number of transitions between healthcare settings for patients [64] but have proven to decrease mortality rates [65, 66]. Little is known, though, as to how centralization has affected patients’ experiences with healthcare services. However, as transitions in healthcare settings have increased, patients’ needs for coordination of healthcare have also arisen, and patients express that the current situation of coordination leaves room for improvement [67, 68]. Although earlier research has shown that interventions in transitions in healthcare settings can improve the patient experience, these studies all focused on a specific type of population and/or on patient experience as a secondary outcome [25–28]. Furthermore, systematic evaluation of patients’ experiences of quality in healthcare transitions following interventions for improvement have been hindered by lacking availability of reliable and valid measures [69]. This affects the results of this scoping review, reducing the comparability between effects of interventions. We suggest addressing assessment of patient-experienced quality in healthcare transitions collaboratively in an international context in the future to enable homogeneous measurement and evaluation of interventions. By studying adult patients’ experiences in general and as a primary outcome we get a better understanding of how interventions can be used on a broader scale. The results of this scoping review suggest that one solution to lessening the burden of transitions for patients – regardless of their illness or disease – could be to implement some form of service to support navigation of the healthcare system. However, due to heterogeneous measurement methods with questionable responsiveness we cannot draw any conclusions as to which interventions are most effective. As healthcare transitions are complex phenomena, we expect that complex interventions with multiple components that are adapted to their context are most efficient [70, 71]. Although care navigators are valued and increase care efficiency [38, 40, 44, 46, 55, 61], it may also be advantageous to reform healthcare systems, so you do not have to be an educated care navigator to be able to access and use healthcare services [72].

We planned this scoping review to have high sensitivity (see protocol at OSF | The Exercise First Research Program) and thus initiated the screening process with a large number of studies. Due to this process, we also had low specificity in our search strategy for the current review. However, this was part of the iterative process which is part of the scoping review method [30], and we would still argue that we have identified the most relevant studies for the aim of this scoping review. It may be seen as a limitation though, that we were seven reviewers. To address the risk of low interrater reliability as a consequence of this, we had several calibration meetings. Thus, we have taken adequate measures to reduce the impact of this potential limitation. Furthermore, we find that we have included more relevant studies than similar reviews [29] and most likely have not excluded relevant reports. Our systematic rigour in all aspects of this review is a strength to the interpretation of our study findings.

The results of this scoping review should not be the only basis for implementing an intervention for patients transitioning between healthcare settings. It is important to take evidence regarding i.e., hospital readmissions or mortality into consideration when choosing which intervention to implement, because even though all interventions seem to help improve patient experience, that might not be the case for other important factors, such as hospital readmissions or mortality. Earlier research suggests that some types of interventions have a significantly higher risk of hospital readmissions than others when patients transition between healthcare settings [26]. Future research investigating the effect of various interventions on both patient experience and other factors, such as mortality and hospital readmissions, could benefit from determining which intervention has the overall best outcomes for patients and hereby which intervention would be most optimal to implement in praxis.

## Supplementary Information

Supplementary Material 1.

Supplementary Material 2.

Supplementary Material 3.

## Data Availability

The datasets used and/or analysed during the current study are available from the corresponding author on reasonable request.
