# Supplementary material for: Identification of interventions to improve patient experienced quality of care in transitions between healthcare settings: a scoping review
Source: BMC Health Serv Res. 2024 Sep 30;24:1155. doi: 10.1186/s12913-024-11609-5 (PMC11443735; doi:10.1186/s12913-024-11609-5)
Supplement: Supplementary file 2 — Supplementary Material 2. [file 12913_2024_11609_MOESM2_ESM.docx]

# Search

The search syntaxes were planned to be run on November 25^th^ 2021, but re-scheduled to December 7^th^ 2021 and re-run on May 27^th^ 2024:

|  | **MEDLINE Ovid (Ovid MEDLINE(R) ALL)** |
| --- | --- |
| 1 | "continuity of patient care"/ or patient discharge/ or patient handoff/ or patient transfer/ or retention in care/ or transitional care/ |
| 2 | *"Delivery of Health Care, Integrated"/ |
| 3 | (care adj2 continu*).ab,kf,ti. |
| 4 | (care adj2 across adj5 sectors).ab,kf,ti. |
| 5 | (care adj2 ?cross adj5 sector*).ab,kf,ti. |
| 6 | (inter* adj2 sector* adj2 care).ab,kf,ti. |
| 7 | (integrat* adj care).ab,kf,ti. |
| 8 | (transition* adj2 care).ab,kf,ti. |
| 9 | (coordinat* adj3 care).ab,kf,ti. |
| 10 | 1 or 2 or 3 or 4 or 5 or 6 or 7 or 8 or 9 |
| 11 | exp Patient Satisfaction/ |
| 12 | (patient* adj1 experience*).ab,ti. |
| 13 | (patient* adj1 perspective*).ab,ti. |
| 14 | (patient* adj2 view*).ab,kf,ti. |
| 15 | (patient* adj2 attitude*).ab,kf,ti. |
| 16 | (patient* adj2 satisf*).ab,kf,ti. |
| 17 | (patient* adj2 involvement*).ab,kf,ti. |
| 18 | (user* adj2 perspective*).ab,kf,ti. |
| 19 | (user* adj2 view*).ab,kf,ti. |
| 20 | (user* adj2 involvement*).ab,kf,ti. |
| 21 | (user* adj2 attitude*).ab,kf,ti. |
| 22 | (user* adj2 satisf*).ab,kf,ti. |
| 23 | (user* adj2 involvement*).ab,kf,ti. |
| 24 | (people* adj1 experience*).ab,ti. |
| 25 | (people* adj1 perspective*).ab,ti. |
| 26 | (people* adj2 view*).ab,kf,ti. |
| 27 | (people* adj2 attitude*).ab,kf,ti. |
| 28 | (people* adj2 satisf*).ab,kf,ti. |
| 29 | (people* adj2 involvement*).ab,kf,ti. |
| 30 | 11 or 12 or 13 or 14 or 15 or 16 or 17 or 18 or 19 or 20 or 21 or 22 or 23 or 24 or 25 or 26 or 27 or 28 or 29 |
| 31 | 10 and 30 |
| 32 | Limit 31 to yr=”2000-Current” |
| 33 | Limit 32 to yr=”all child (0 to 18 years)” |
| 34 | Limit 33 to yr=”all adult (19 plus years)” |
| 35 | 33 not 34 |
| 36 | 32 not 35 |
|  | **EMBASE Ovid** |
| 1 | exp integrated health care system/ |
| 2 | exp transitional care/ |
| 3 | (care adj2 continu*).ab,kf,ti. |
| 4 | (care adj2 across adj5 sectors).ab,kf,ti. |
| 5 | (care adj2 ?cross adj5 sector*).ab,kf,ti. |
| 6 | (inter* adj2 sector* adj2 care).ab,kf,ti. |
| 7 | (integrat* adj2 care).ab,kf,ti. |
| 8 | (transition* adj2 care).ab,kf,ti. |
| 9 | (coordinat* adj3 care).ab,kf,ti. |
| 10 | 1 or 2 or 3 or 4 or 5 or 6 or 7 or 8 or 9 |
| 11 | *patient satisfaction/ or *patient attitude/ |
| 12 | (patient* adj1 experience*).ab,ti. |
| 13 | (patient* adj1 perspective*).ab,ti. |
| 14 | (patient* adj2 view*).ab,kf,ti. |
| 15 | (patient* adj2 attitude*).ab,kf,ti. |
| 16 | (patient* adj2 satisf*).ab,kf,ti. |
| 17 | (patient* adj2 involvement*).ab,kf,ti. |
| 18 | (user* adj2 perspective*).ab,kf,ti. |
| 19 | (user* adj2 view*).ab,kf,ti. |
| 20 | (user* adj2 involvement*).ab,kf,ti. |
| 21 | (user* adj2 attitude*).ab,kf,ti. |
| 22 | (user* adj2 satisf*).ab,kf,ti. |
| 23 | (user* adj2 involvement*).ab,kf,ti. |
| 24 | (people* adj1 experience*).ab,ti. |
| 25 | (people* adj1 perspective*).ab,ti. |
| 26 | (people* adj2 view*).ab,kf,ti. |
| 27 | (people* adj2 attitude*).ab,kf,ti. |
| 28 | (people* adj2 satisf*).ab,kf,ti. |
| 29 | (people* adj2 involvement*).ab,kf,ti. |
| 30 | 11 or 12 or 13 or 14 or 15 or 16 or 17 or 18 or 19 or 20 or 21 or 22 or 23 or 24 or 25 or 26 or 27 or 28 or 29 |
| 31 | 10 and 30 |
| 32 | Limit 31 to child <unspecified age> |
| 33 | Limit 32 to adult <18 to 64 years> |
| 34 | Limit 33 to aged <65+ years> |
| 35 | 32 not 33 not 34 |
| 36 | 31 not 35 |
| 37 | Limit 36 to yr=”2000-current” |
|  | **EBSCOhost CINAHL** |
| 1 | (MH "Continuity of Patient Care+") |
| 2 | (MM "Transitional Care") |
| 3 | TI(integrat* N3 care*) OR AB(integrate* N3 care*) |
| 4 | TI(transition* N2 care*) OR AB(transition* N2 care*) |
| 5 | TI(care N1 across N5 sector*) OR AB(care N1 across N5 sector*) |
| 6 | TI(care N2 ?cross N5 sector*) OR AB(care N2 ?cross N5 sector*) |
| 7 | TI(inter* N2 sector* N2 care) OR AB(inter* N2 sector* N2 care) |
| 8 | TI(care N2 continu*) OR AB(care N2 continu*) |
| 9 | TI(coordinat* N3 care* ) OR AB(coordinat* N3 care* ) |
| 10 | 1 or 2 or 3 or 4 or 5 or 6 or 7 or 8 or 9 |
| 11 | (MH "Patient Satisfaction+") |
| 12 | (MM "Patient Attitudes") |
| 13 | TI(patient* N1 experience*) OR AB(patient* N1 experience*) |
| 14 | TI(patient* N1 perspective*) OR AB(patient* N1 perspective*) |
| 15 | TI(patient* N1 view*) OR AB(patient* N1 view*) |
| 16 | TI(patient* N2 satisf*) OR AB(patient* N2 satisf*) |
| 17 | TI(patient* N2 attitude*) OR AB(patient* N2 attitude*) |
| 18 | TI(patient* N2 involvement*) OR AB(patient* N2 involvement*) |
| 19 | TI(user* N2 experience*) OR AB(user* N2 experience*) |
| 20 | TI(user* N2 perspective*) OR AB(user* N2 perspective*) |
| 21 | TI(user* N2 view*) OR AB(user* N2 view*) |
| 22 | TI(user* N2 involvement*) OR AB(user* N2 involvement*) |
| 23 | TI(user* N2 satisf*) OR AB(user* N2 satisf*) |
| 24 | TI(user* N2 attitude*) OR AB(user* N2 attitude*) |
| 25 | TI(people* N1 experience*) OR AB(people* N1 experience*) |
| 26 | TI(people* N1 perspective*) OR AB(people* N1 perspective*) |
| 27 | TI(people* N1 view*) OR AB(people* N1 view*) |
| 28 | TI(people* N2 satisf*) OR AB(people* N2 satisf*) |
| 29 | TI(people* N2 attitude*) OR AB(people* N2 attitude*) |
| 30 | TI(people* N2 involvement*) OR AB(people* N2 involvement*) |
| 31 | 11 or 12 or 13 or 14 or 15 or 16 or 17 or 18 or 19 or 20 or 21 or 22 or 23 or 24 or 25 or 26 or 27 or 28 or 29 or 30 |
| 32 | 10 and 31 |
| 33 | Limiters: Published Date 20000101- |
