# Supplementary material for: Identification of interventions to improve patient experienced quality of care in transitions between healthcare settings: a scoping review
Source: BMC Health Serv Res. 2024 Sep 30;24:1155. doi: 10.1186/s12913-024-11609-5 (PMC11443735; doi:10.1186/s12913-024-11609-5)
Supplement: Supplementary file 3 — Supplementary Material 3. [file 12913_2024_11609_MOESM3_ESM.pdf]

Appendix 1- Extraction template

General information

- Title
- Author(s)
- Year of publication
- Country of origin (list of countries to cross of which)

Characteristics of included studies

- Methods
  - Aim/purpose/research question(s)
  - Study design (list of all the different study designs to cross of which)
  - Intervention/implemented service/change in procedure
- Participants
  - Population description
  - Inclusion criteria
  - Exclusion criteria
  - Number of participants
- Outcomes/phenomena of interest
  - What were they?
  - How were they collected? (list of collection methods to cross of)
  - How were they measured or analyzed? (list of measurement/analysation methods to cross of)
- Key findings
  - Qualitative findings
  - Quantitative findings
